# The first six years of surveillance in pediatric and neonatal intensive care units in Turkey

**DOI:** 10.1186/s13756-015-0074-3

**Published:** 2015-08-25

**Authors:** Emine Alp, Tülay Orhan, Cemile Atalay Kürkcü, Safiye Ersoy, Mary-Louise McLaws

**Affiliations:** Faculty of Medicine, Department of Infectious Diseases and Clinical Microbiology, and Infection Control Committee, Erciyes University, Kayseri, Turkey; Faculty of Medicine, Faculty of Medicine, Infection Control Committee, Erciyes University, Kayseri, Turkey; School of Public Health and Community Medicine, UNSW Medicine, The University of New South Wales, Level 3 Samuels Building, Sydney, NSW 2052 Australia

**Keywords:** Surveillance, Healthcare, Associated, Infection, Antibiotic, Consumption, Multiple, Resistance

## Abstract

**Background:**

Patients in resourced-limited neonatal and pediatric intensive care units (NICU and PICU) are vulnerable to healthcare associated infections (HAI). We report the incidence of HAI, multidrug resistant microorganisms (MDROs) and the pattern of antibiotic usage in the first six years of a surveillance program in a teaching hospital in Turkey.

**Methods:**

Between 2007 and 2012 surveillance data for HAI, MDROs and antibiotic usage were collected from the infection control department, pathology, hospital admissions and pharmacy. In 2009 hand hygiene auditing was introduced. Hand sanitizer usage was expressed as liters per 1000 patient-days. Antibiotic usage was presented as defined daily doses (DDD). Evidence of change in the incidence of HAI was tested using Poison regression modeling.

**Results:**

The rate of gram negative MDRO in PICU increased significant between 2007 and 2012 (IRR 1.5, *P* = 0.033) but remained unchanged in NICU (*P* = 0.824). By 2012 ceftriaxone prescribing in PICU had decreased while carbapenem prescribing increased by 80 %. In NICU carbapenem decreased by 42 % and betalactam decreased by 29 %. Hand hygiene compliance significantly improved in PICU (IRR 1.9, *p* < 0.001) and NICU (IRR 2.2, *p* < 0.001) but compliance remained modest after three years with inconsistent levels across the 5 moments.

**Conclusion:**

The early years of our infection control program highlights the endemicity of HAI and MDROs in our NICU and PICU. The consistent pattern of antibiotic usage, endemic MROs in PICU and modest hand hygiene clearly provide strategic focuses for intervention.

## Introduction

Healthcare associated infections (HAI) remain a major safety problem for vulnerable patients in pediatric and neonatal intensive care units (ICUs) worldwide [1–3]. In low to middle income countries where infection control programs are resource-poor or limited, pediatric ICU (PICU) and neonatal ICU (NICU) patients are especially vulnerable to HAIs [1]. In high-income countries evidence-based infection control programs have successfully reduced HAIs rates [4]. However, the feasibility of successful implementation of programs outside high resourced countries is questionable given the limited data from low and middle income countries [2]. Turkey is a middle-income country [5] with rising HAI rates and in 2000 the newly formed Turkish Society of Hospital Infection and Control developed guidelines and introduced training for infection control nurses and doctors. The Ministry of Health responded in 2005 by mandating public hospitals provide routine reports on infection control activities and in 2006 a new national surveillance system was rolled out [6]. We report the incidence of HAI over the first 6-years of a surveillance program in a typical tertiary PICU and NICU in Turkey.

## Methods

### Description of institution and infection control program

Erciyes University Hospital is a tertiary referral facility in the Central Anatolian region of Turkey. Institutional approval was given for reporting the data. The pediatric hospital is collocated on the adult hospital campus with 154,680 pediatric admissions and 1,653 ICU admissions annually. Surgery is rarely performed with approximately 11 pediatric or neonatal patients undergoing surgery per year. There are 2 level-III ICUs; a 10-bed pediatric ICU (PICU) for patients aged 1 month to 18 years and an 18-bed neonatal ICU (NICU) with babies as small as <750 g. Bed spacing is >1.5 m and there is 1 sink provided for every 2-beds. PICU has 1 isolation room for every 5-beds and in NICU there is 1 isolation room for every 9-beds. In 2006 an organized infection control program introduced a policy that one trained infection control doctor and one certified infection control nurses (ICN) were to be employed for each unit as a full-time equivalent (FTE) staff. Since 2006, all patients admitted to ICU have been routinely screened for vancomycin resistant enterococci and carbapenem resistant Enterobacteriaceae. Surveillance definitions were applied in accordance with the Centers for Disease and Control (CDC) [4]. From 2009 a multimodal hand hygiene education promotion campaigns was introduced that included alcohol based hand rub (ABHR) at every bedside, chlorhexidine containing soap and hand hygiene compliance audits with feedback. Trained ICNs audit healthcare workers’ (HCWs) compliance with *My Five Moments* using the World Health Organization audit tool and audit in accordance with the guidelines [7]. Monthly and quarterly HAI and hand hygiene results are reported to the infection control committee.

### Data collection

Between 2007–2012 surveillance for HAI included active surveillance by the ICN who also validated all laboratory HAI notifications. Surveillance data are collected routinely as a quality and safety activity of the Hospital. These data were collected from the infection control department as well as device utilization and device-associated infection rates for central line associated bacteremia (CLABSI), catheter associated urinary tract infections (CAUTI), incidence of multidrug resistant microorganisms (MDRO) and hand hygiene compliance. Hospital admissions provided the number of patient days, occupancy rate per year, length of stay and the 24-h nurse-to-patient ratio. The nursing workload was measured by the 24-h nurse-to-patient ratio divided by the total number of nurses during the 24-h period using the patients’ census for the day. Consumption of alcohol based hand rub (ABHR) was provided by pharmacy and expressed as liters per 1000 patient-days. Pharmacy provided antimicrobial use (reported as defined daily dose [DDD] and is the average maintenance dose per day for each drug) normalized per 1000 patient-days in accordance with World Health Organization (WHO Collaborating Centre for Drug Statistics Methodology, 2012). The most widely used antibiotics examined for usage were: ceftriaxone, beta-lactam/beta-lactamase inhibitors, carbapenem, aminoglycoside, glycopeptide, linezolid, metronidazole, quinolones, colistin, tigecycline, fluconazole. A microorganism was defined as MDRO if it provided resistant to more than three classes of antibiotics; aminoglycosides, antipseudomonal penicillins, carbapenems, cephalosporins, beta-lactam/beta-lactamase inhibitor, quinolones, colistin, tigecycline [7]. The most prevalent MDRO included *Acinetobacter baumannii*, *Pseudomonas aeruginosa*, extended spectrum beta-lactamase (ESBL) *Klebsiella pneumoniae* and ESBL *Esherichia coli*, methicillin resistant *Staphylococcus aureus* (MRSA) and ampicillin resistant *Enterococcus.* Pathogens were identified using Vitek-2 automated method and interpreted according to Clinical Laboratory Standards Institute Guidelines: CLSI. Mean inhibitory concentrations were performed using a standard method for antimicrobial susceptibility testing in accordance with Twenty-fourth informational supplement M100-S18 (Villanova, PA, USA, 2008).

### Analysis

The difference in the mean length of stay, with ± one standard deviation (SD), was calculated using ANOVA. Frequencies and 95 % CIs were established separately for hand hygiene compliance in PICU and NICU from 2009 to 2012. Total HAI rates expressed per 1000 patient-days and VAP, CLABSI and CAUTI were expressed per 1000 device-days, with 95 % CIs for 2007 to 2012. Exact binomial method around a proportion was used to test for change in incidence. All three specific HAIs were aggregated to examine whether small number of infections in each of the three types prevented detection in the change in rates. On examination of the precision of the estimates, using 95 % CIs, precision was not warranted to one decimal place and all decimals were rounded up at 0.6 and only rates <1 were present to one decimal point. Changes in the percentage point difference in hand hygiene rates from 2009 to 2012 were calculated. The effect of the introduction of the *My five moments for hand hygiene* campaign on total compliance for each of the five hand hygiene indications and HAI was estimated using incidence rate ratio (IRR) produced from a Poisson generalized linear model (GLM) which was chosen because total HAI had a Poisson distribution. IRR could not be established where rates were zero. The first year of observation was used as the baseline for measuring change in IRRs over the following years. HAI were statistically rare events resulting in the inability for the effect of hand hygiene on specific HAIs to be modeled using either GLM or negative binomial regression. Hand hygiene compliance rates were also reported for the magnitude of change described in percentage points (PP). Alpha was set at the 5 % level. All analysis was undertaken using SPSS version 21 (IBM, Armonk, NY).

## Results

### Patient acuity

The average number of patient-days for 2007–2012 in NICU was 4,610 (range 3,994 to 5,681). In PICU the average number of patient-days for 2007–2012 was 3,333 (range 3,050 to 3,516). The mean length of stay in NICU was 30.2 days (SD ± 11.1) in 2007 and increased to 38.5 days (SD ± 14.2) by 2012 (*P* < 0.0001). The mean length of stay in PICU was 28.3 days (SD ± 19.0) in 2007 and increased to 29.3 (SD ± 13.3) by 2012 (*P* < 0.0001). In 2007 the ratio of nurse-to-patient admissions in NICU was 0.9:1 and steadily increased to reach 1.3:1 by 2012. In PICU the ratio of nurse-to-patient admissions remained at 1:1 with the exception of 2008 and 2009 when the ratio increased to 1.3:1 and 1.6:1 respectively before returning to 1:1 thereafter.

### Specific healthcare associated infections

The rate of CAUTI in NICU remained stable between 2007 (9 per 1000 catheter-days, 95 % CI 0.2–52) and 2012 (4 per 1000 catheter-days, 95 % CI 0.1–22) (IRR 0.43, *P* = 0.670). In PICU the CAUTI rate remained stable between 2007 (8 per 1000 catheter-days, 95 % CI 3–15) and 2012 (9 per 1000 catheter-day, 95 % CI 5–15) (IRR 1.3, *P* = 0.622).

The CLABSI rate in NICU did not change significantly (*P* = 1.0) between 2007 (0 per 1000 line-days, 95 % CI 0–4) and 2012 (0 per 1000 line-days, 95 % CI 0–11). In PICU the CLABSI rate remained stable between 2007 (6 per 1000 catheter-days, 95 % CI 3–13) and 2012 (8 per 1000 catheter-days, 95 % CI 5–13) (IRR 1.2, *P* = 0.615).

The rate of VAP in NICU remained unchanged between 2007 and 2012 ranging from 10 (95 % CI 7–14) per 1000 device-days in 2007 to 7 (95 % CI 4–10) per 1000 device-days (IRR 0.71, *P* = 0.814). In PICU the VAP rate remained unchanged from 9 (95 % CI 6–14) per 1000 device-days in 2007 to 11 (95 % CI 8–16) per 1000 device-days in 2012 (IRR 1.2, *P* = 0.412).

### Total healthcare associated infections

The rate of aggregated HAIs in NICU remained stable over the first six years of surveillance with the rate at 20 per 1000 patient-days in 2007 and 19 per 1000 patient-days in 2012 (IRR 0.93, *P* = 0.957) (Table 1). The total HAI rate in PICU in 2007 was 39 per 1000 patient-days and declined significantly in 2010 (IRR 0.61, *P* < 0.001) and in 2012 (IRR 0.65, *P* = 0.002) (Table 1).

**Table 1:** Healthcare associated infections for Neonatal and Pediatric ICUs between 2007 and 2012

Year	Neonatal ICU	Pediatric ICU
2012	19 [16–23] (108/5681)	25 [20–31] (88/3460)
	IRR = 0.93, 0.957	IRR = 0.65, 0.002
2011	18 [14–21] (86/4792)	24 [19–29] (83/3516)
	IRR = 0.87, 0.925	IRR = 0.60, <0.001
2010	23 [19–28] (106/4522)	24 [19–30] (83/3457)
	IRR = 1.10, 0.925	IRR = 0.61, 0.001
2009	20 [16–24] (84/4238)	32 [26–38] (109/3411)
	IRR = 0.97, 0.980	IRR = 0.81, 0.117
2008	22 [17–27] (87/3994)	38 [31–45] (116/3050)
	IRR = 1.1, 0.967	IRR = 0.97, 0.804
2007	20 [17–25] (91/4434)	39 [33–47] (122/3106)
	IRR = 1 (reference year)	IRR = 1 (reference year)

Rate/1000 patient-days [95 % CI] (HAI/patient-days), Incidence rate ratio (IRR), *P*-value

### Multiple drug resistant microorganisms

The rate of MDROs in NICU remained unchanged for *A. baumanni* starting at 2.26 per 1000 patient-days in 2007 and remained steady at 2.82 per 1000 patient-days in 2012 (IRR 0.8, 95 % CI IRR 0.4–2, *P* = 0.58)*, K.pneumonia* at 1.13 and 0.7 per 1000 patient-days in 2007 and 2012 respectively (IRR 0.6, 95%CI IRR 0.2–2.5, *P* = 0.483) and *P.aeroginosa* at 1.58 and 0.7 per 1000 patient-days in 2007 and 2012 respectively (IRR 0.4, 95%CI IRR 0.1–1.4, *P* = 0.198). When all GNB were aggregated the rate in NICU from 2007 and 2012 (IRR 0.9, 95%CI IRR 0.5–2, *P* = 0.824).

The rates in PICU remained unchanged for *A. baumanni* starting at 7.41 per 1000 patient-days in 2007 and remained steady at 5.49 per 1000 patient-days in 2012 (IRR 0.74, 95 % CI IRR 0.5–1.4, *P* = 0.335) and *K.pneumonia* also remained steady at 3.54 and 1.73 per 1000 patient-days between 2007 and 2012 respectively (IRR 0.5, 95 % CI IRR 0.2–1.4, *P* = 0.159). *P.aeroginosa* decreased from 5.15 per 1000 patient-days in 2007 to 2.02 per 1000 patient-days by 2012 (IRR 0.4, 95 % CI IRR 0.2–1 *P* = 0.039). When all GNB MRDOs were aggregated the rate in PICU significantly decreased from 18.3 per 1000 patient-days in 2007 to 11.8 per 1000 patient-days by 2012 (IRR 0.7, 95 % CI IRR 0.5–1, *P* = 0.033).

The MRSA rate in NICU in 2007 was 0 per 1000 patient-days and remained stable with the exception of 2008 when 2 patients acquired MRSA infection (0.5 per 1000 patient-days, *P* = 1.0). The MRSA rate in PICU in 2007 was 2 per 1000 patient-days and dropped significantly to 0 per 1000 patient-days in 2012 (*P* < 0.05).

### Antimicrobial daily dose usage

**Fig. 1:**
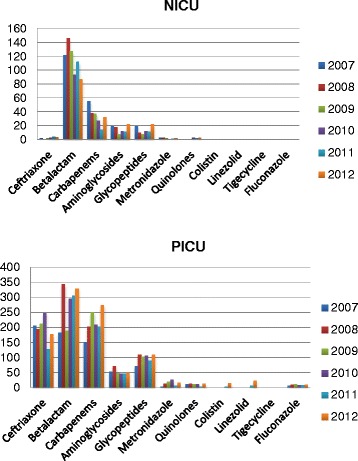
Antibiotic gram usage in Neonatal and Pediatric ICUs between 2007-2012

In NICU betalactam antibiotics (range 87 to 146 g) were more commonly prescribed while carbapenems, aminoglycosides and glycopeptides were prescribed less frequently (Fig. 1).

By 2012 betalactam prescribing had decreased by 29 % (122 g to 87 g) and carbapenem consumption decreased by 42 % (55.4 g to 32 g). In PICU the most common antibiotics were betalactams, carbapenems and ceftriaxone while aminoglycoside and glycopeptides were less common. In PICU by 2012, ceftriaxone usage had fallen 14 % (207 g in 2007 to 178 g in 2012). Increases in usage in PICU were marked by an 80 % increase in carbapenem usage (152 g in 2007 to 273 g in 2012) and 44 % increase in betalactam usage (183 g in 2007 to 329 g in 2012). PICU and NICU infrequently prescribed metronidazole, quinolone, colistin, linezolid, tigecycline and fluconazole.

### Hand hygiene by moments

Total compliance in hand hygiene significantly improved in both ICUs after the introduction in 2009 of the hand hygiene program (Tables 2 and 3). Compliance with moment 5, after touching the patient surroundings, did not improved in either NICU or PICU. By 2012 compliance in the NICU increased significantly for moment 1 (IRR 1.5, 65.6 %, *p* = 0.001), moment 2 (IRR 2.0, 79 %, *p* = 0.001) and moment 4 (IRR 1.4, 76 %, *p* = 0.006) (Table 2). By 2012 compliance in PICU increased significantly for moment 1 (IRR 2.4, 50 %, *p* < 0.001), moment 2 (IRR 4.6, 48 %, *p* < 0.001) and moment 4 (IRR 1.5, 67 %, *p* = 0.008) (Table 3). NICU had higher compliance than PICIU for moment 1 by 15 percentage points (PP) in 2012, 31PP higher for moment 2 and 9PP higher for moment 4.

**Table 2:** Neonatal ICU Hand hygiene compliance by Moments between 2009 and 2012

Year	Five Moments % complied [95 % CI] (Observed/Total opportunities) Incidence Rate Ratio (IRR), *P*-value					
	Moment 1 Before patient contact	Moment 2 Before a procedure	Moment 3 After procedure/risk of exposure to body fluid	Moment 4 After patient contact	Moment 5 After contact with patient environment	Total Moments
2012	65.6 [60.6–70.2] (259/395) IRR = 1.5, 0.001	78.7 [68.7–86.6] (70/89) IRR = 2.0, 0.001	14.9 [9.4–22.1] (20/134) IRR = 1.5, 0.338	76.4 [71.7–80.6] (281/368) IRR = 1.4, 0.006	33.4 [27.9–39.3] (93/278) IRR = 0.67, 0.782	57.2 [54.4–59.9 %] (723/1264) IRR = 2.2, <0.001
2011	62.5 [58.1–66.7] (318/509) IRR = 1.4, 0.004	59.4 [51.1–67.4] (88/148) IRR = 1.5, 0.046	25.8 [18.4–34.4] (32/124) IRR = 2.6, 0.016	65.2 [60.5–69.7] (281/431) IRR = 1.3, 0.018	55.7 [47.8–63.4] (93/167) IRR = 0.59, 0.731	58.1 [55.4–60.7] (801/1379) IRR = 2.9, <0.001
2010	57.0 [53.3–60.7] (414/726) IRR = 1.3, 0.031	56.3 [50.6–61.9] (174/309) IRR = 1.4, 0.062	17.4 [12.8–22.9] (41/235) IRR = 1.7, 0.150	68.8 [65.4–72.1] (525/763) IRR = 1.2, 0.050	37.7 [30.3–45.5] (63/167) IRR = 1 (reference year)	56.3 [54.2–58.4] (1217/2160) IRR = 4.7, <0.001
2009	44.6 [37.8–51.5] (95/213) IRR = 1 (reference year)	39.0 [28.0–50.7] (30/77) IRR = 1 (reference year)	10.0 [4.4–18.8] (8/80) IRR = 1 (reference year)	56.1 [48.8–63.1] (111/198) IRR = 1 (reference year)	-	43.0 [38.8–47.1] (244/568) IRR = 1 (reference year)

**Table 3:** Pediatric ICU Hand hygiene compliance by Moments between 2009 and 2012

Year	Five Moments % complied [95%CI] (Observed/Total opportunities) Incidence Rate Ratio (IRR), *P*-value					
	Moment 1 Before patient contact	Moment 2 Before a procedure	Moment 3 After procedure/risk of exposure to body fluid	Moment 4 After patient contact	Moment 5 After contact with patient environment	Total Moments
2012	50 [45–55] (231/461) IRR = 2.4, <0.001	48 [40–55] (88/185) IRR = 4.6, <0.001	28 [23–33] (97/345) IRR = 4.2, 0.001	67.5 [63–71] (353/523) IRR = 1.5, 0.008	43 [39–47] (251/585) IRR = 1.6, 0.749	49 [46–51] (1020/2099) IRR = 1.9, <0.001
2011	46 [41–52] (179/385) IRR = 2.2, 0.001	52 [45–58] (124/239) IRR = 5.0, <0.001	39 [34–45] (120/304) IRR = 5.9, <0.001	74 [69.9–78.3] (324/436) IRR = 1.7, 0.001	48 [42–54] (139/291) IRR = 1.4, 0.807	53 [51–56] (886/1655) IRR = 2.4, <0.001
2010	43 [39–47] (315/733) IRR = 2.0, 0.002	46 [42–51] (217/469) IRR = 4.5, <0.001	24 [21–28] (132/541) IRR = 3.7, 0.002	67 [63–71] (467/696) IRR = 1.5, 0.008	43 [42–50] (251/544) IRR = 1 (reference year)	43 [42–45] (1296/2983) IRR = 2.1, <0.001
2009	21 [14–31] (21/99) IRR = 1 (reference year)	10 [4–20] (7/68) IRR = 1 (reference year)	7 [2–14] (6/90) IRR = 1 (reference year)	45 [35–55] (48/107) IRR = 1 (reference year)	-	22 [18–27] (82/364) IRR = 1 (reference year)

**Fig. 2:**
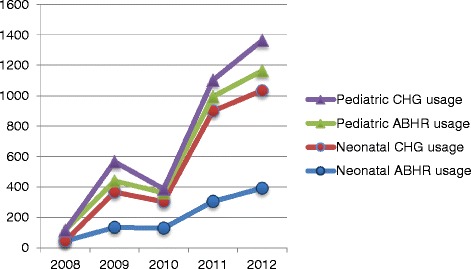
Liter usage of Alcohol Based Hand Rub (ABHR) and Chlorhexidine (CGH) containing soap in Neonatal and Pediatric ICUs between 2008-2012

Total compliance in 2012 in NICU was 9PP higher than PICU and this was reflected in the ABHR usage in NICU that was three times higher than in PICU (Fig. 2).

## Discussion

Intrinsic risk factors, such as an immature immune system and severity of illness, and extrinsic factors, such as poor health services infrastructure, invasive devices and the medication associated with these devices, increase the risk of HAI acquisition in patients admitted to NICU and PICU [3, 8]. In low to middle income countries factors associated with the persistence of HAI in NICU and PICU are unlike those in high income countries, such as a lack of crucial elements of an infection control program, a legal framework for establishing and enforcing infection control programs and finances [2]. Other risk factors unique to low and middle income healthcare settings is overcrowded wards, poor nurse-to-patient ratio, patient malnutrition and severity of illness [2]. Since 2006 some of the essential components of the infection control programs in our ICUs are similar to high resourced countries and includes the development of infection control procedures, surveillance, education and monthly feedback to ICU staff on their HAI prevention performance. Yet, despite comparable device utilization rates to those reported in the USA the device-associated HAIs in both our ICUs were 2–20 times higher than the rates in USA [9]. Rosenthal et al. also reported a higher incidence of VAP in academic hospitals in low to middle income countries [3]. Smulders et al. demonstrated that infection prevention bundles are effective in neonatal and pediatric patients rate of CLABSI and VAP [10]. Yet, our intractable CAUTI, CLABSI and VAP rates remain unexplained and may be due to a lack of institutionalized compliance by our PICU or NICU with each of the bundle items. The drop in HAI is most likely in response to the aggregation of statistically rare events and not a result of infection prevention. Similarly, our inability to identify a significant change in MDROs in PICU except when we aggregated all GNB suggests that the sample size is possibly a factor. Our academic hospital receives patients transferred with severe illness. Important intrinsic factors contributing to HAI were not evaluated and compliance with prophylaxis was also not evaluated. However, poor antibiotic prescribing and poor hand hygiene compliance may be two important causal contributors.

Antibiotics are the most frequently used medicines in neonatal and pediatric ICUs. Only consumption in grams was measured so judgment of antibiotic over-prescribing in our tertiary referral hospital with admissions and transfers of patients with severe disease can only be suggested from the trends in consumption. Although we expect antibiotics are commenced empirically, consumption of the most commonly prescribed antibiotics in NICU appeared to have declined. The carbapenem resistant *A.baumannii* in PICU was endemic between 2009 and 2012 and unlike carbapenem usage globally [11] there was a conspicuous increase, 80 %, in carbapenem prescribing during this period. In NICU, staff carbapenem prescribing patterns decreased by 42 % over the same period and may have been in response to a peak in carbapenem resistant *A.baumannii* in 2011. Colistin prescribing is rare in our ICUs unlike elsewhere [11]. The persistence of GNB MDROs, specifically *A.baumannii, K.pneumonia and P. aeroginosa,* and our patterns of repeated and prolonged prescribing need to be challenged to urgently address habitually poor prescribing practices by our ICU physicians [12–14]. Even in the absence of decolonization practices, such as chlorhexidine body wash [15], a modest improvement in our hand hygiene rates has kept the transmission of MRSA infections low [16]. We did not evaluate the association between antibiotic use and resistance patterns or reduction in infection due to change in antibiotic prescribing.

The introduction of a hand hygiene program in 2009 resulted in a 15PP improvement in total compliance by NICU staff and a 27PP improvement by PICU staff. Compliance with moment 1, moment 2 and moment 4 improved 21PP, 40PP and 20PP respectively in NICU while poor compliance for moments 3 and 5 reduced the overall compliance rate. PICU staff performed moments 2, 3 and 5 poorly while the improvements in Moments 1 and 4 by 29PP and 22PP respectively suggests a modest change in behavior. Glove use for moments 2 and 3 may explain our failure to achieve important behavioral change. However, even with the availability of ABHR and monthly compliance feedback to staff the final compliance in both ICUs was still low. The method of direct observation for monitoring hand hygiene compliance usually results in some degree of Hawthorne Effect, therefore the usual compliance rates may in fact be lower than the rate we are reporting.

In Turkey, national infection control programs commenced nearly 50 years after other European countries. Despite our late start and limited resources, we have demonstrated that our surveillance activities have continued. Countries with limited resources have constraints in common such as, hospital infrastructure, overcrowding, understaffing, poor infection control program funding and adherence to guidelines. The barriers to further progression includes the continued high workload demonstrated by our very low nurse-to-patient ratio ranging 0.6:1 to 0.9:1 in PICU and 0.5:1 to 0.6:1 in NICU. High patient loads may be an important barrier to hand hygiene practice [17]. Low nurse-to-patient ratio significantly increases the risk for HAI with a much as 30 % of HAI possibly being avoided when nurse-to-patient ratio is maintained at >2.2 nurses to one patient [18]. Two contributing factors to the endemic HAI rates and poor hand hygiene compliance in our ICUs may include nursing staff caring for >1 patient during night shifts and the prolonged night shift, routinely 16-h. These two factors have been found to adversely impact a clinician’s ability to perform at their very best [18, 19]. Innovative behavior change programs [20] may have a more immediate benefit on HAI rates given we may not be able to remedy the nurse-to-patient ratio in the near future. *Acinetobacter* species readily acquire resistance due to, in part, its excellent biofilm-producing ability that enables it to survive in hospital environments [21]. Any breakdown in environmental cleaning will contribute to a persistent endemicity, such as we have witnessed.

Our study provides an insight into the early phase of infection control programs in a middle-income country although we acknowledge that there are limitations to interpretations of our study that includes the collection of certain data retrospectively, absence of reasons for prescribing and patient demographic characteristics. Given any improvements in infrastructure will be slow to occur, a middle-income healthcare system such as ours needs to look towards a more effective use of existing resources, effecting governmental prioritization of HAI prevention programs and urgent changes to antibiotic prescribing practices.
